# Reply to Comment on “Malfitano, A.M. et al. Virotherapy as a Potential Therapeutic Approach for the Treatment of Aggressive Thyroid Cancer” *Cancers* 2019, *11*, 1532

**DOI:** 10.3390/cancers12020281

**Published:** 2020-01-23

**Authors:** Anna Maria Malfitano, Sarah Di Somma, Nella Prevete, Giuseppe Portella

**Affiliations:** Dipartimento di Scienze Mediche Traslazionali, Università Federico II Napoli, URT “Genomic of Diabetes” of Institute of Experimental Endocrinology and Oncology “G. Salvatore”, National Council of Research (CNR), 80131 Naples, Italy; annamaria.malfitano@unina.it (A.M.M.); sarah_ds@hotmail.it (S.D.S.); nella.prevete@unina.it (N.P.)

Our work is focused on the future clinical use of oncolytic viruses (OVs) for the treatment of aggressive thyroid carcinomas. Therefore, we provide a brief description of the overall use of OVs in the clinic. Rigvir is among the few OVs that have already been used for the treatment of patients, and studies describing its effects have been briefly commented and cited in our text [1].

The comment of Dr. Alberts aims to clarify the actual state of the oncolytic ECHO-7 virus Rigvir and its approval as a therapeutic opportunity. The comment properly declares that Rigvir has been approved in former Soviet Union countries such as Latvia in 2004, Georgia in 2015, Armenia in 2016, and Uzbekistan in 2018 [2].

In the sentence stated in our publication: “This virus has not met the required standard regulation for European or U.S. approval, further studies are in progress to better evaluate its therapeutic potential”, we did not doubt the efficacy of Rigvir. Indeed, many pre-clinical and clinical studies have been performed. However, it is clear that Rigvir use needs to be supported by further and updated preclinical and clinical studies as also argued in the paper “The advent of oncolytic virotherapy in oncology: The Rigvir^®^ story” by Dr. Alberts, where “the results will be confirmed and updated by modern clinical studies”. In our work, it is not stated that the use of Rigvir was rejected by European or U.S. agencies, but likely that more detailed studies are required to propose Rigvir for their approval. Overall, if the readers require more specific information regarding Rigvir, proper references have been included by us and we are sure that more focused reviews such as those of Dr. Alberts, might clarify any “unclear” sentences.
